# Jejunal perforation and upward migration of double J stents during the cystoscopic procedure: a case report and review of literature

**DOI:** 10.11604/pamj.2022.42.56.33727

**Published:** 2022-05-19

**Authors:** Faisal Ahmed, Qasem Alyhari, Saif Ghabisha, Saleh Al-wageeh, Ebrahim Al-shami, Menawar Dajenah, Waleed Aljbri, Fawaz Mohammed

**Affiliations:** 1 Urology Research Center, Al-Thora General Hospital, Department of Urology, Ibb University of Medical Science, Ibb, Yemen,; 2 Department of General Surgery, Ibb University of Medical Science, Ibb, Yemen,; 3 Department of Urology, School of Medicine, 21 September University, Sana'a, Yemen,; 4 Department of Orthopedy, Ibb University of Medical Science, Ibb, Yemen

**Keywords:** Double-j stent, complications, jejunal perforation, upward migration, case report

## Abstract

While double J (DJ) stenting is common worldwide in the urological procedure, it may associate with severe and catastrophic complications. Penetration of the jejunum and upward migration of double J (DJ) stents during cystoscopic DJ stent procedure are rare complications with few reported cases in the literature. We present a 65-year-old male presented with acute renal failure and peritonitis one week after failed cystoscopic removal of DJ stents. Radiographic investigations showed upward migration of the right DJ stent and a total displacement of the left DJ stent to the peritoneal cavity with peritonitis, bladder perforation, and jejunal injuries. The right DJ stent was removed via the ureteroscopic procedure. Then, open abdominal surgery was performed to remove the left DJ stent and repair the injured bladder wall and jejunal segment. In conclusion, synchronous upward DJ stent migration and peritoneal DJ stent malposition with jejunal and bladder injuries are rare and severe complications of the cystoscopic DJ stent procedure. The treatment should be performed depending on the time of diagnosis, nature of the injury, and general clinical conditions of the patient.

## Introduction

Double J (DJ) stents facilitate the urine drainage from the renal pelvis to the urinary bladder in various urological conditions such as urinary tract obstruction, stenosis, and fistula [1]. DJ stents are a routine and straightforward process in urological practice and may perform via antegrade or retrograde approach [1,2]. Infection, stent irritation, stent migration, encrustation, and a forgotten stent are all risks associated with this procedure [3]. While, upward DJ stent migration, bladder perforation, jejunal injury, and peritonitis due to the peritoneal cavity's perforation are rare complications [3,4]. Synchronous jejunal perforation and upward migration as complications of double J stents removal have not been reported to our knowledge. Hence, we present a rare case of DJ stenting complications, including right upward migration of DJ stent and a total displacement of left DJ stent to the peritoneal cavity with bladder perforation, jejunal injury, and peritonitis with a review of previously published cases of peritoneal perforation during cystoscopic procedure.

## Patient and observation

**Figure 1:**
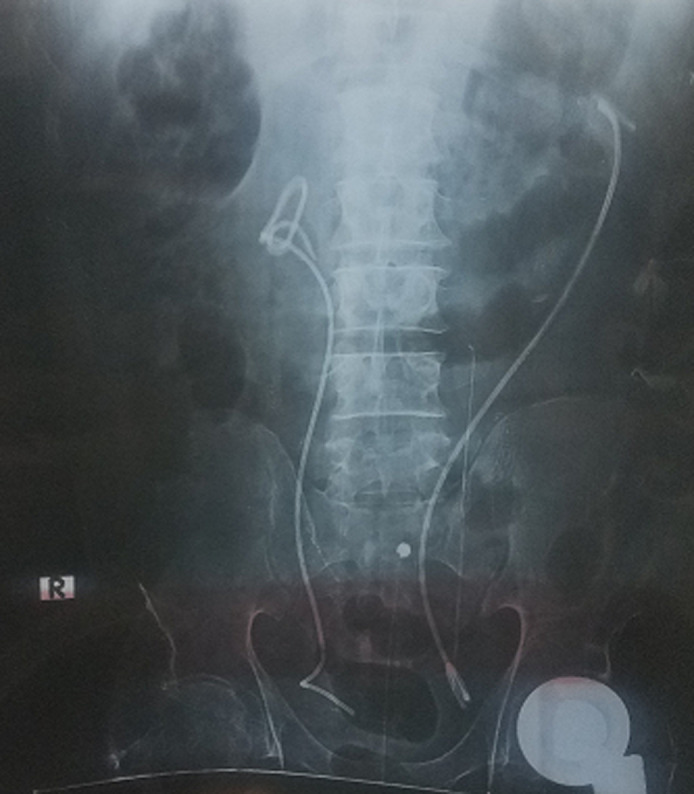
plain radiography X-ray showing both DJ stents at first insertion

**Patient information:** a 65-year-old man presented to our emergency department with a history of decreased urine output, fever, and abdominal pain in the last week. The patient underwent a cystoscopy, bilateral DJ stent insertion, and urethrotomy due to urethral stricture and severe bilateral hydronephrosis by another urologist in another center (Figure 1). The same urologist tried to remove both DJ stents one week later, but was unsuccessful. The patients' condition worsened, and he was admitted to the intensive care unit for three days. During admission, the patient developed acute renal failure and peritonitis. The patient is a case of diabetes mellitus on oral hypoglycemic agents and had a history of the right lower extremity amputation nine years ago.

**Clinical findings:** the patients' vital sign was blood pressure: 110/70 mmHg, respiratory rate: 16 respirations per minute, the pulse rate: 61 beats per minute, and oral temperature: 38.5°C. He was pale and had severe and generalized abdominal tenderness on palpation.

**Figure 2:**
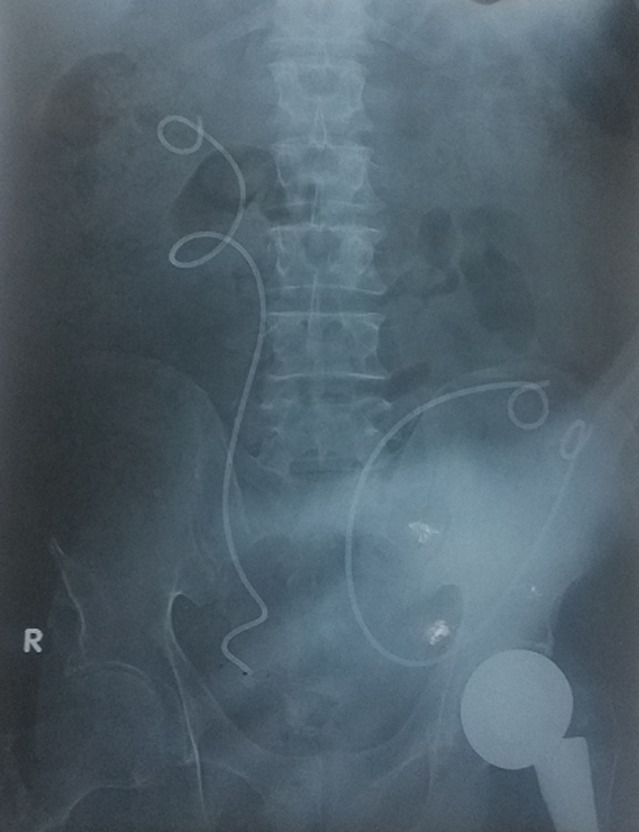
plain radiography X-ray showing both DJ stents after failed removal

**Figure 3:**
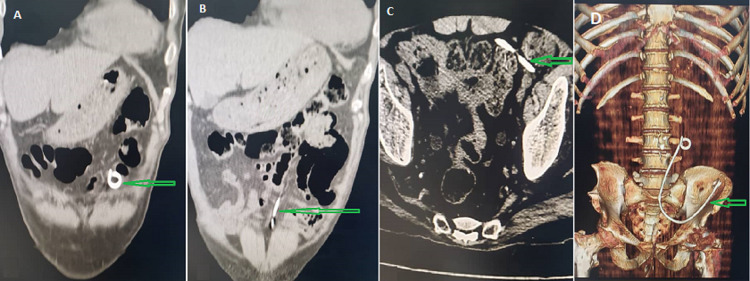
abdominal CT scan showing: A) distal coil of double J stent in the peritoneal cavity (arrow); B) double J stent in the peritoneal cavity (arrow); C) proximal coil of double J stent in the peritoneal cavity (arrow); D) helical computed tomography shows the DJ stent's total displacement in the peritoneal cavity (arrow)

**Diagnostic assessment:** urine analysis showed microscopic hematuria and pyuria (15-20 RBC / HPF and 15-20 / HPF puss cells). The blood investigation revealed serum creatinine: 7.4 mg/dl, blood urea nitrogen: 105 mg/dl, white blood cells: 35 x 10^3^/ml with an increase in absolute neutrophil, and hemoglobin: 11.6 g/dl. The results of all other blood tests were within the normal range. On plain radiography, the right DJ stent migrated upward (the distal coil of the DJ stent was at the level of the sacroiliac joint upon insertion), and the left DJ stent was totally forced and inserted into the abdominal cavity (Figure 2). An abdominal pelvic computed tomography (CT) scan was requested and showed the total migration of the left DJ stent to the abdominal cavity with suspected penetration of the bladder and jejunum by a proximal coil of the DJ stent and moderated free fluid in the abdominal cavity in favor of peritonitis (Figure 3).

**Therapeutic interventions:** firstly, urgent hemodialysis and broad-spectrum antibiotics (Cefepime 1 g every 12 hours and Metronidazole 500 mg every 12 hours) were started. After stabilization of the patient, we recommended removing the DJ stents. The right DJ stent was removed via ureteroscopy. The left ureteral orifice was not identified due to multiple false passages and multiple traumas to the bladder mucosa. After dissections with the patients' families, removing the stent via open surgical exploration was decided.

**Figure 4:**
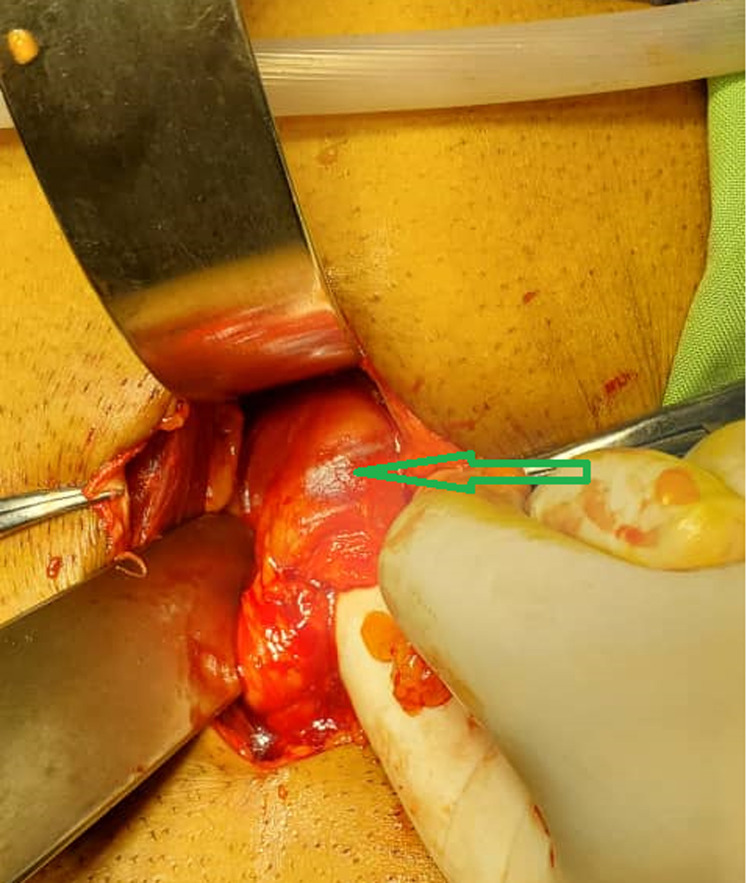
intraoperative photos showing the double J stent in the peritoneal cavity (arrow)

**Figure 5:**
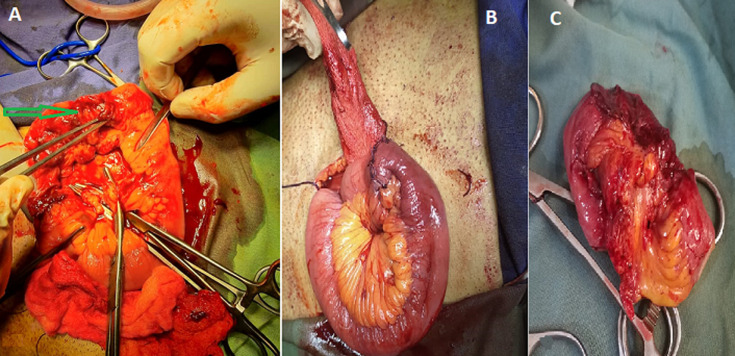
intraoperative images showing: A) the jejunal perforation site (arrow); B) the jejunal repaired; C) part of the jejunum resected

During surgery, the DJ stent was identified to perforate the jejunum and caused trauma to the other bowel lope with severe adhesion between the bladder wall and the bowel content (Figure 4). Intraperitoneal rupture of the bladder wall was also identified. After removing the DJ stent, the injured part of the jejunum was resected, and the general surgery team performed an intestinal anastomosis (Figure 5). The site of the perforated urinary bladder was repaired with vicryl 3/0. Then the drain was inserted into the abdominal cavity, and the facia and the skin were closed with nylon 2/0.

**Follow-up and outcome:** antibiotic therapy was maintained for one week (Cefepime 1 g 2-fold by day and Metronidazole 500 mg 2-fold by day). On the fourth postoperative day, the renal function test was normal. On the 15^th^ postoperative day, the patient left our hospital in good condition. The patient's general condition was good at five months of follow-up without significant complications.

**Patient perspective:** the patient was happy with the successful outcome of the surgery.

**Informed consent:** written informed consent was obtained from the patient for participation in our study.

## Discussion

The ureteral stenting was first used in 1967 and then applied to advance endourological approaches [5]. Currently, DJ stents are widely used to treat upper urinary tract stones, obstructive uropathy, ureteral strictures, ureteral injuries, and tumor compression [1]. Complications of DJ stents include hematuria, irritative bladder symptoms, infection, encrustation, and fragmentation. These issues are minor and manageable [2,6].

Upward migration of a DJ stent is rare, with incidences ranging from 1.2% to 8.2% reported in the literature [6]. Total abdominal displacement of a DJ stent is uncommon and has only been reported in a few cases [6,7]. Additionally, iatrogenic jejunal injury caused by DJ stenting is rare and usually occurs after gastrointestinal endoluminal intervention procedures [7]. Our patient had two complications of DJ stent; the first one was the right upward migration of DJ stent, and the second was left total DJ stent displacement into the peritoneal cavity with jejunal perforation, peritonitis, and bladder wall injuries which was the first reported case to our knowledge.

Several authors have mentioned successfully managing upward-migrated DJ stents via ureteroscopy procedures, such as Tan *et al*. and Bagley *et al*. [8,9]. In our case, the right DJ stent was successfully removed by the ureteroscopic procedure. Many factors influence stent migration, including the shape, length, and material of the stents. The significant factors associated with upward DJ stent migration are the short stent, an angle in the distal part of the stent <180 degrees, and the upper pole DJ stent insertion [1]. The patients' characteristics that could lead to stent malposition include low body mass index, kyphoscoliosis, and renal movement during respiration [10,11]. Furthermore, surgeon factors may contribute to stent migration, such as poor positional play and erroneous deployment [5,11]. In our patients, instead of surgeon experience, patient comorbidities such as diabetes mellitus, severe hydronephrosis, urethral stricture, and restricted mobility due to lower extremity amputation may be an additional risk factor for those complications.

The second complication was the total abdominal displacement of the left DJ stent, bladder perforation, and jejunal injury. Malposition of a DJ stent can occur when the bladder mucosa is inflamed, allowing the guidewire to perforate the bladder wall, resulting in a false passage and faulty DJ stent placement [7]. Urinary leakage into the peritoneal cavity, peritonitis, bowel injuries, bladder perforation are a complication of peritoneal perforation during this procedure. Those complications require a high suspicion index, especially in patients with symptoms of peritonitis in the postoperative period [5]. In our patient, it is possible that the mechanism of peritoneal perforation was caused by an incorrectly placed DJ stent outside of the ureteral orifice and considerable damage to the bladder mucosa when the urologist tried to remove it. However, the technical details of our patient procedure were not accessible, and another urologist from a nearby hospital performed this procedure. Recently reported cases and their management of DJ stent displaced into the peritoneal cavity were summarized in Table 1 [3,4,7,12-18].

**Table 1:** recently reported cases of DJ stent displaced into the peritoneal cavity and the management

No	Author, year	Age (years), gender	Time of stenting	Complication	Management
1	Wall, 2008	84, F	8 years	Duodenal perforation	Open nephrectomy and duodenal repair
2	Sanjay Prakash, 2020	59, M	3 months	Duodenal perforation	Laparoscopic nephrectomy and duodenal repair
3	Quadri, 2020	59, M	3 months	Duodenal perforation	Laparoscopic retrieved the DJ stent to the ureter and duodenal repair
4	Rhee, 2013	78, F	3 months	Retroperitoneum perforation	Percutaneous antegrade nephroscopic DJ stent retrieval
5	Turri, 2015	59, M	8 months	Intraperitoneal perforation	Open surgical exploration and remove the DJ stent
6	Vijayaraghavan, 2020	55, M	3 weeks	Descending colon perforation	Replacing the stent with a new stent
7	Ivica, 2009	46, F	5 years	Right mesocolon perforation	Nephrectomy, repair of perforation
8	Kholis, 2021	50, M	5 years	Pelvic cavity perforation	Transurethral cystolithotripsy
9	Katz	62, F	2 weeks	Pelvic cavity perforation	Left antegrade nephrostomy insertion
10	KAR, 1984	72, F	18 months	Ureterocolic fistulas	Left nephrectomy and left to right ureteropyelostomy

DJ: double J; M: male; F: female

Risk factors for cystoscopic bladder perforation are chronic urinary retention, urethral stricture, diverticula, chronic inflammation, tumors, radiation cystitis, and excessive alcohol consumption [19]. In our case, urethral stricture resulting in chronic urinary retention and multiple false passages during the cystoscopic procedure may be risk factors for bladder perforation. The main symptom of peritonitis appeared to be severe abdominal discomfort and rapid, deep, and continual pelvic pain. Vast urinary leaking into the peritoneal cavity may lead to abdominal compartment syndrome, respiratory distress, and anuria [17]. Our patient experienced progressively worsening peritoneal irritation and renal failure, typical of urinary peritonitis.

The treatment of these complications depends on the time of diagnosis, nature of the injury, and general clinical conditions of the patients [20]. The management options include laparoscopy, cystoscopic DJ stent removal or repositioning, and open laparotomy. Laparoscopy is highly valuable in this case with dense adhesions because gentle dissection can be performed under direct vision, allowing stent removal and repair of the injured bowel segment. If a uretero-enteric fistula is present, a uretero-ureterostomy, fistulous tract excision, and reconstructive repair of the bowel segment may be required. If intraperitoneal bladder rupture and peritonitis are evident, this may need urgent open laparotomy [1,20]. Synchronous jejunal and bladder perforation were reported during Nd-YAG laser coagulation for refractory superficial bladder cancer by Ruiz-Tovar *et al*. The patient was treated with resection of the perforated jejunal loop and primary anastomoses and bladder wall repaired [20]. A similar procedure was performed in our case. Additionally, the open surgical approach was the best option for our patient due to the lack of a center with laparoscopic facilities in our city, high suspension of peritonitis, and our patient's financial situation, which limited us to referring hem to another center.

This report's final recommendation is to avoid blind DJ stent insertion or removal, especially in acute or inflammatory conditions. The optimal way to avoid DJ stent complications is to have the DJ stent placed under fluoroscopic control and perform postoperative radiography [8]. Maintaining a high index of suspicion, particularly in patients with postoperative peritonitis symptoms, is essential to avoiding future catastrophic complications.

## Conclusion

Synchronous upward DJ stent migration and peritoneal DJ stent malposition with jejunal and bladder injuries are rare and severe complications of the cystoscopic DJ stent procedure. The treatment should be performed depending on the time of diagnosis, nature of the injury, and general clinical conditions of the patient.
